# Pedal to the metal: Cities power evolutionary divergence by accelerating metabolic rate and locomotor performance

**DOI:** 10.1111/eva.13083

**Published:** 2020-09-25

**Authors:** Lacy D. Chick, James S. Waters, Sarah E. Diamond

**Affiliations:** ^1^ Department of Biology Case Western Reserve University Cleveland OH USA; ^2^ Hawken School Gates Mills OH USA; ^3^ Department of Biology Providence College Providence RI USA

**Keywords:** contemporary evolution, Formicidae, metabolism, plasticity, temperature, thermal performance curve, urban heat island

## Abstract

Metabolic rates of ectotherms are expected to increase with global trends of climatic warming. But the potential for rapid, compensatory evolution of lower metabolic rate in response to rising temperatures is only starting to be explored. Here, we explored rapid evolution of metabolic rate and locomotor performance in acorn‐dwelling ants (*Temnothorax curvispinosus*) in response to urban heat island effects. We reared ant colonies within a laboratory common garden (25°C) to generate a laboratory‐born cohort of workers and tested their acute plastic responses to temperature. Contrary to expectations, urban ants exhibited a higher metabolic rate compared with rural ants when tested at 25°C, suggesting a potentially maladaptive evolutionary response to urbanization. Urban and rural ants had similar metabolic rates when tested at 38°C, as a consequence of a diminished plastic response of the urban ants. Locomotor performance also evolved such that the running speed of urban ants was faster than rural ants under warmer test temperatures (32°C and 42°C) but slower under a cooler test temperature (22°C). The resulting specialist–generalist trade‐off and higher thermal optimum for locomotor performance might compensate for evolved increases in metabolic rate by allowing workers to more quickly scout and retrieve resources.

## 1 INTRODUCTION

Metabolic rate—the energy used by an organism per unit time—is a fundamental property of life and is highly sensitive to changes in the environment (Chown & Gaston, 1999). For many ectothermic species, metabolic rate is strongly influenced by temperature, rising gradually from a lower limit up to an optimum and declining rapidly to an upper limit (Dell, Pawar, & Savage, 2011). As local and global temperatures rise through human‐mediated environmental change, many ectotherms are expected to exceed their optimum temperature for metabolic rate (Dillon, Wang, & Huey, 2010). However, this expectation ignores the ability of organisms to mount compensatory responses to warming. Contemporary adaptive evolution of temperature‐relevant traits is emerging as a key compensatory mechanism for a number of taxa (De Meester, Stoks, & Brans, 2018). Yet the potential for contemporary evolution of metabolic rate under rising temperatures is only starting to be explored (*e.g*., Moffett, Fryxell, Palkovacs, Kinnison, & Simon, 2018; Padfield, Yvon‐Durocher, Buckling, Jennings, & Yvon‐Durocher, 2016; Pilakouta et al., 2020).

Although far from a universal pattern, multiple biogeographic comparative studies find lower metabolic rates of ectotherms from warmer climates compared with those from cooler climates when tested at the same temperature (i.e., the metabolic cold adaptation hypothesis, Addo‐Bediako, Chown, & Gaston, 2002; DeLong et al., 2018; Gaston et al., 2009). This countergradient variation is interpreted as an adaptation to cold temperatures, enabling growth, development, and reproduction in cold climates with limited growing seasons (Du, Warner, Langkilde, Robbins, & Shine, 2010; Terblanche, Clusella‐Trullas, Deere, Van Vuuren, & Chown, 2009). However, with recent climatic warming, the focus is shifted toward the evolution of metabolic rate under warmer conditions. The expectation of countergradient variation is the same, but the mechanism is different. In this case, adaptation to high temperatures mitigates the negative effects of heat stress (Pilakouta et al., 2020).

In addition to the directionality of the effect being unsuitable for inferences of responses to climatic warming (i.e., adaptation to cold rather than to heat), such biogeographic comparative studies necessarily focus on long‐diverged populations, making it difficult to assess the potential for metabolic rate to evolve in response to temperature over contemporary timescales. As an exception, recent observational and experimental work in guppies found evidence of contemporary evolution of metabolic rate in concert with variation in predation regime and abiotic environmental changes (Auer, Dick, Metcalfe, & Reznick, 2018), but this study did not examine temperature per se. Further, an experimental evolution study on phytoplankton found that selection for tolerance of high temperatures was associated with a reduction in metabolic rate (Padfield et al., 2016). Although metabolic rate was not the direct target of selection in this experiment, selection on thermal tolerance led to a concomitant reduction in metabolic rate and yielded a pattern of countergradient variation.

Perhaps the best evidence of contemporary evolution of metabolic rate in response to temperature comes from work in fish systems. Moffett et al. (2018) examined populations of the western mosquitofish introduced from Texas, USA, into springs in California, USA, and the North Island of New Zealand within the last 100 years. The springs varied considerably in temperature, and the researchers found evidence of countergradient variation in the allometry and temperature dependence of routine metabolic rate that served to limit the costs associated with increased metabolic rate in warm environments. Based on the mosquitofish generation time, metabolic rate evolved over a period of 180 generations. Likewise, Pilakouta et al. (2020) examined populations of threespine stickleback that either inhabited unaltered water bodies or those that had been artificially warmed through excess hot water runoff from residential geothermal heating over the last 50 to 70 years. The researchers found the evolution of lower thermal sensitivity of standard metabolic rate resulting in significantly lower metabolic rate at warmer laboratory acclimation temperatures. The stickleback have a generation of 1 year, meaning that metabolic rate evolved over 50 to 70 generations in this system.

Although contemporary evolution of metabolic rate in response to warming is clearly possible, it is also useful to consider the interplay of shifts in metabolic rate with other metabolism‐related traits. Because metabolism involves the use of energy by organisms, the ability to acquire energy is a key metabolism‐related trait (Marshall, Dong, McQuaid, & Williams, 2011; Sibly, Brown, & Kodric‐Brown, 2012). As temperatures rise, demands on energy inputs of ectotherms typically also increase (Buckley, Hurlbert, & Jetz, 2012). This relationship has led some researchers to forecast the “metabolic meltdown” of ectothermic organisms under climatic warming, as many organisms will be incapable of acquiring sufficient food resources to meet the increased metabolic demand of warmer climates (Huey & Kingsolver, 2019). Here again, however, it is important to consider such predictions in context of potential compensatory evolutionary responses of resource acquisition traits under climatic warming. Indeed, locomotor performance traits, while serving a range of critical functions from finding mates to avoiding predators, are also important for finding and retrieving food resources (e.g., Miles, 2004) and tend to be highly sensitive to temperature (Bennett, 1990). Thus, evolution of locomotor performance traits could aid in understanding the response of metabolic rate to warming.

Cities provide a unique opportunity to explore the potential for contemporary evolution of thermal physiological and performance traits in response to warming. Through the proliferation of roads, sidewalks, and buildings, urban heat islands warm intra‐city areas by several degrees Celsius compared with adjacent or nearby undeveloped areas (Manoli et al., 2019). Although there are currently only a handful of studies on the evolution of urban thermal physiology and performance, the results so far indicate a range of co‐ and countergradient responses to urban warming. Tüzün, Op de Beeck, Brans, Janssens, and Stoks (2017) uncovered countergradient variation for development time in urban versus rural populations of damselfly larvae wherein urban larvae developed more slowly due to the extended growing season in warm urban habitats compared with the shorter growing season in cool rural habitats. In this same study, cogradient variation was found for survival, with urban larvae having higher survival at all temperatures. McLean, Angilletta, and Williams (2005) found similar results for chitinolytic fungi that exhibited a range of co‐ and countergradient growth rate responses to urbanization. Exploring responses at the sub‐cellular level, Brans, Stoks, and De Meester (2018) found countergradient variation for oxidative stress responses in water fleas such that urban populations evolved lower baseline activity levels of enzymes important for stress physiology. Yet the authors detected cogradient variation for energy budgets in which urban populations had higher energy budgets at all temperatures. Thus, with the range of co‐ and countergradient thermal physiological responses observed in response to urban heat islands, it is very much an open question of whether the expectation of countergradient variation for metabolic rate will be met and how other potential metabolism‐related traits such as locomotor performance will respond.

Here, we used the acorn ant, *Temnothorax curvispinosus*, to explore the potential for contemporary evolution of metabolic rate and locomotor performance in response to urban warming. This ant species inhabits acorns and other small cavities in both nonurban “rural” forests and forest patches within urbanized areas (Diamond, Chick, Perez, Strickler, & Martin, 2017). Acorn ants are also a good candidate system to explore urban evolution of metabolism as we already have evidence of adaptive evolution of higher heat tolerance in urban acorn ants (Diamond, Chick, Perez, Strickler, & Martin, 2018). Further, like many ant species (Hurlbert, Ballantyne, & Powell, 2008), acorn ant locomotor performance is also strongly dependent on temperature such that warmer developmental temperatures and warmer test temperatures lead to faster running speed (Maclean, Penick, Dunn, & Diamond, 2017). In ants, running speed is important both for quickly locating resources (Hölldobler & Wilson, 1990; Lach, Parr, & Abbott, 2010; Pearce‐Duvet, Elemans, & Feener, 2011) and returning resources to the nest (Leonard & Herbers, 1986), particularly for subordinate, generalist scavenger species like *T. curvispinosus* (Fellers, 1987). In this study, we tested multiple urban and rural populations in Cleveland, Ohio, USA (42°N latitude; WorldClim mean annual temperature 10°C, Fick & Hijmans, 2017) for metabolic rate as direct indicator of energy use and running speed, a locomotor performance trait as a potential proxy of resource acquisition, or energy gain. The urban heat island effect in acorn ant microhabitats at this specific location is appreciable, exceeding 4°C, on average during the growing season (Diamond, Chick, Perez, Strickler, & Martin, 2018).

We used a single‐temperature laboratory common garden rearing design with multiple test temperatures to quantify evolutionary divergence between urban and rural populations and acute thermal plasticity. Ant colonies were reared in the laboratory at 25°C for 3 months until a new cohort of ant workers was generated, after which time metabolic rate and running speed were assessed. We measured whole‐colony resting metabolic rate at the laboratory rearing temperature of 25°C, and we re‐measured the same colonies after a 2‐hr exposure to 38°C. On a different set of colonies, we tested individual worker ant running speed at three test temperatures: 22°C, 32°C, and 42°C. We expected that warmer test temperatures would lead to plastic effects of increased metabolic rate and running speed across urban and rural populations. We further expected that urban populations would exhibit an evolved lower metabolic rate and/or faster running speed as compensatory responses to urban heat islands compared with rural populations.

## 2 MATERIALS AND METHODS

Our study comprised two main experiments, one that focused on metabolic rate and another that focused on running speed. The experiments were performed in the same geographic location (Cleveland, OH, USA), but in different years (2017 for metabolic rate and 2018 for running speed). In both experiments, we examined evolutionary divergence between urban and rural acorn ant source habitats and acute thermal plasticity in response to different test temperatures. Ideally, the metabolic rate and running speed assays would have been performed on the same colonies to best understand the relationships between these traits, though this was not possible owing to logistical constraints. Importantly, however, we were able to test colonies from the same general area, if not exactly the same sites and populations, across the two experiments (Table S1). This site overlap provided some continuity between the results for metabolic rate and running speed and allowed us to compare the phenotypic patterns of metabolic rate and running speed responses to temperature.

### 2.1 Colony collections and laboratory rearing

For the metabolic rate experiment, we collected whole colonies of the acorn ant, *T. curvispinosus*, from urban and rural habitats in Cleveland, OH, USA (42°N latitude), between May and June 2017. We used percent developed impervious surface area (ISA) from the National Land Cover Database (Yang et al., 2018) as the criterion for designating urban and rural sites. Urban sites were defined as those with ISA values between 40% and 61%, and rural sites were defined as those with ISA values of 0%. Mean ISA values for each site were calculated with a 120 m buffer using the focal statistics tool in the spatial analyst toolbox in ArcGIS 10.4. We collected a total of 41 colonies, including 20 colonies from 4 urban sites and 21 colonies from two rural sites (Table S1). Note that each rural site contained two sub‐sites that were approximately 1–1.5 km apart from one another. Our statistical model results (see below) were very similar whether we modeled these sub‐sites separately or grouped together. To ensure our results and interpretations were conservative, we opted to group the sub‐sites together for each rural site location. We attempted to include colonies of comparable sizes with respect to the number of queens, workers, and brood, both within and among urban and rural source habitats. Although we still had variation in colony size within each source habitat, colony size did not differ statistically between source habitat (Table S2).

We maintained colonies in environmental growth chambers (Panasonic MIR154) under a constant 25°C temperature regime with a 14:10 L:D photoperiod for 12–13 weeks to ensure a new cohort of workers was produced (Diamond, Chick, Perez, Strickler, & Martin, 2018). Queen life span often exceeds 5 years, whereas worker life span is approximately 30 days under these conditions (Keller, 1998; Modlmeier, Foitzik, & Scharf, 2013). The minimum of 3 months that colonies were held in the laboratory ensured that individuals were produced in the laboratory (F1 worker ants) without prior field developmental acclimation effects. Colonies were housed individually in 120‐ml plastic cups with ad libitum resource tubes of sugar water (25% sucrose solution), tap water, and dead mealworms.

We followed a similar collection scheme and laboratory rearing protocol for the running speed experiment. A total of 53 colonies were collected, including 26 from two rural sites and 27 from five urban sites, between July and August 2018 (Table S1). Note that the rural sites had the same sub‐site structure as described above for the colonies assessed for metabolic rate; however, some urban sites differed from those used to measure metabolic rate. For each colony, 10 individual workers were assessed for running speed. Here again, there was variation in colony size, which can affect running speed in ants (Leonard & Herbers, 1986); however, colony size was not significantly different between urban and rural source habitats (Table S2).

### 2.2 Experimental design and response assays

#### 2.2.1 Metabolic rate experiment

We measured whole‐colony resting metabolic rates using flow‐through respirometry (Lighton, 2008). Metabolic rate testing occurred between August 28 and September 4, 2017. We transferred all individuals (queens, workers, and brood) of a single colony from their acorn nest into an airtight respirometry chamber with a Delrin base and Plexiglas lid (Waters, Ochs, Fewell, & Harrison, 2017), and we allowed the colony to acclimate to their new enclosure at 25°C in an environmental growth chamber (DigiTherm DT2‐MP‐47) for 30 min. This experimental design controls for prior field developmental acclimation effects on worker ant metabolism, but does not eliminate potential maternal effects on workers nor the effects of the field environment on colony queens. However, for other physiological traits including heat and cold tolerance, maternal effects on workers are negligible in this system (Martin, Chick, Yilmaz, & Diamond, 2019). More generally, while maternal effects across a diverse range of study organisms are nonzero, they are considerably weaker than effects due to additive genetic variation (Moore, Whiteman, & Martin, 2019). Further, field effects on queens and maternal effects on workers are likely to be small given the long (minimum 3 month) laboratory acclimation period prior to testing of metabolic rate. Finally, although our experimental design was unable to avoid potential direct effects of the field environment on queens as they were field‐caught and lived for the duration of the experiment, the biomass of queens (1–3 per colony) is much smaller than the biomass of laboratory‐born workers (on average, 34 workers per colony, in this experiment). Thus, while we are unable to fully exclude a potential role for maternal effects, we think they are unlikely to be the sole or even dominant explanation for our results.

**FIGURE 1:**
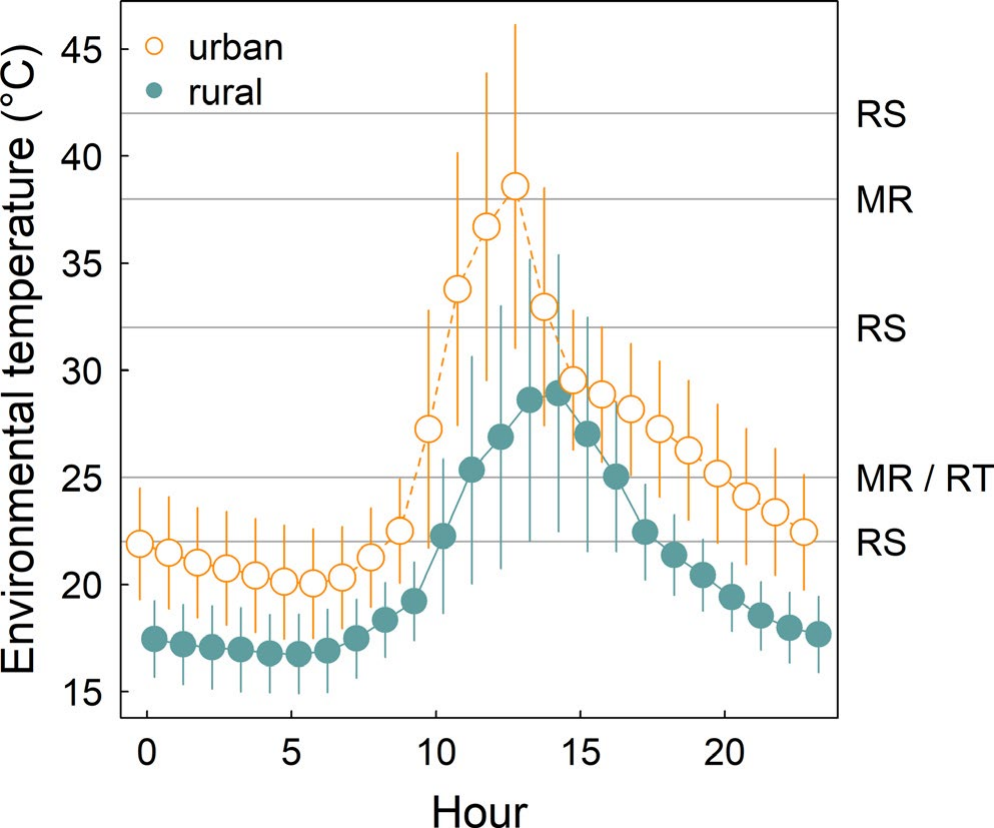
Environmental temperature profiles within acorn ant nest microsites in urban and rural habitats. Mean hourly temperature (points) ±1 *SD* from June 16, 2016, through July 15, 2016, in Cleveland, Ohio, USA (data from Diamond, Chick, Perez, Strickler, & Martin, 2018). Horizontal gray lines represent the test temperatures for metabolic rate (MR) and running speed (RS). The laboratory rearing temperature (RT) to produce a new cohort of laboratory‐born worker ants for both the metabolic rate and running speed experiments is also indicated

We first measured metabolic rate at 25°C. We then exposed the colony to 38°C for 2 hr and measured metabolic rate again. Both temperatures are experienced by urban and rural acorn ants in the field. Though, notably, whereas the 25°C test temperature is frequently experienced by both urban and rural ants, the rural ants only experience 38°C just over 6% of the days during the growing season compared with 67% of the days for the urban ants (Figure 1). We conducted all respirometry measurements within environmental growth chambers (DigiTherm DT2‐MP‐47) to minimize fluctuations in temperature. The dry CO_2_‐free air supply for our push‐mode system was provided from an ultra‐zero air compressed gas cylinder. We regulated air flow rate at 50 ml/min using an Omega mass flow controller (FMA 5506, 0–50 ml/min) and measured using a flow meter within a FoxBox (Sable Systems International, SSI). We measured carbon dioxide concentration using an infrared CO_2_ analyzer in differential mode (LiCor 7000). To control for analyzer drift, the system was baselined for 2 min, then the airflow from the chamber was measured for at least 15 min, and then a second baseline was measured for an additional 2 min. Airflow was switched between baseline and colony chambers using a multiplexer (MUX, SSI), and air was dried prior to measuring CO_2_. We collected data using an analog to digital converter (UI‐2, SSI) and recorded at 1 Hz in Expedata software (SSI). We report colony metabolic rate as mean CO_2_ (ppm) over the recording interval.

Our experimental design measures resting metabolic rate, defined as metabolic rate in which the organism's energy output is reduced but which still allows for low levels of activity (Auer, Killen, & Rezende, 2017). In general, *Temnothorax* spp. ants have low activity levels compared with other ant taxa (Charbonneau, Hillis, & Dornhaus, 2015; Herbers & Cunningham, 1983). Most importantly, the 30‐min acclimation period prior to the assessment of metabolic rate allowed the acorn ants to regather their nest, and limited movement of the colony in the chamber during the assessment of metabolic rate. Because we moved whole colonies into chambers designed to mimic acorn ant nest proportions (Herbers & Johnson, 2007), there was no potential for foraging or excavation. When placed in the respirometry chambers, workers briefly walked the perimeter of the chamber, then aggregated brood around the queen, and began tending to brood, indicating a state of rest within the experimental apparatus similar to that of their natural nest.

Nonetheless, we aimed to directly quantify the overall activity levels of acorn ants within the respirometry experimental apparatus and the potential for differences in activity between ants from urban versus rural source habitats. Our access to sensors that measured metabolic rate was limited to the metabolism experiment described above. However, we later performed a follow‐up experiment (in 2018) to assess ant activity within respirometry chambers (using the same Delrin base and Plexiglas lid design as that used in the metabolic rate assessments), though the chambers were not connected to sensors to measure metabolic rate. We assessed activity of colonies (*n* = 8) collected from the same urban and rural sites in Cleveland as those colonies that underwent the metabolic rate testing (Table S1). The colonies were collected concurrently with the colonies used in the running speed trials and were held at 25°C in the laboratory for a minimum of 12 weeks prior the assessment of activity in late November 2018. Whole colonies, with comparable numbers of queens, workers, and brood to the colonies assessed for metabolic rate (Supplementary Information Text, Figure S1), were placed inside respirometry chambers, which were in turn placed inside environmental growth chambers (DigiTherm DT2‐MP‐47) held at 25°C. Ants were allowed to adjust to the chamber environment for 30 min prior to recording activity. Colonies were then exposed to 38°C for 2 hr prior to recording activity a second time. Activity was measured as the number of worker ants actively walking around the respirometry chamber during a 10‐s interval. This process was repeated every 2 min over the course of 20 min, totaling 11 time points per each colony × test temperature. Ants had overall low levels of activity and critically did not exhibit significant differences in worker activity among urban versus rural source habitats. Video analysis of the number of individual workers moving around the respirometry chamber revealed no significant effect of source habitat on this response variable (Supplementary Information Text, Table S3; Figure S2).

For these reasons, we have little evidence that differences in activity between urban and rural source habitats would serve as a potential alternative explanation for evolutionary divergence in metabolic rate. However, even if activity levels were different between urban and rural source habitats, because we reared acorn ants in the laboratory under common garden conditions until a new cohort of workers was produced, any differences in activity level would be strong evidence of evolutionary divergence in this trait. These evolved differences in activity level among source habitats would then simply provide an intermediate mechanism for evolutionary divergence in metabolic rate.

#### 2.2.2 Running speed experiment

Although acorn ants are relatively inactive within the nest environment when they are surrounded by the entire colony (as simulated by the respirometry chamber), they are highly active throughout the foraging landscape when they are isolated from the rest of the colony (Herbers & Johnson, 2007). Therefore, population divergence in locomotor performance when ants are placed in a foraging context could still be possible even though urban and rural ant activity levels are comparable within the nest environment context. For individual worker ants, we assessed locomotor performance by measuring their running speed across multiple test temperatures. We recorded the time (in seconds) for an individual worker ant to move in 20 mm increments from 0 to 100 mm, hereafter “distance intervals.” Ants were gently prodded with a paintbrush as a means to standardize the cue for the running response, though foragers are generally moving anyway outside the nest environment. Ants were tested on tracks consisting of thermal tape covered by a ceramic tile to achieve three different constant test temperatures of 22°C, 32°C, and 42°C. Track temperatures were adjusted via thermostatic control and confirmed with both a thermal image camera (FLIR C2) and bead‐tipped thermocouples (Sper Scientific 800024). Ants were always tested in the same order across temperature treatments from lowest test temperature to highest to avoid order effects of test temperatures on subsequent trials. Ants were given a 2‐hr period with access to water, but not food, prior to the first running speed trial, and the same between subsequent running speed trials. The running speed trials were performed between October 22 and November 18, 2018.

### 2.3 Statistical analyses

#### 2.3.1 Metabolic rate experiment

We fit a linear mixed effects model (REML) using the *lmer* function from the {lmerTest} library in R (Kuznetsova, Brockhoff, & Christensen, 2017; R Core Team, 2019) to examine the effects of source habitat (urban or rural), test temperature (25°C or 38°C), and their interaction on colony metabolic rate. Owing to variation in the number of individuals per colony, which impacts colony‐level metabolic rate (Waters et al., 2017), we included colony mass (g) as a continuous covariate in the model. To meet multivariate normality assumptions, we log_10_‐transformed the colony mass covariate and the response variable, metabolic rate (Waters et al., 2017). Our model also included a hierarchical random effects structure. As we had multiple colonies obtained from the same site (with multiple urban and rural sites in our experimental design), we included site as a random intercept to account for autocorrelation of colonies from the same site. We also included colony identity as a random intercept, nested within site, to account for the repeated measures of metabolic rate on colonies, first at 25°C and again at 38°C. Initially, we attempted to allow the slopes of the relationship between metabolic rate and temperature to vary across colonies by constructing a random slope and intercept model. Although such a model did not converge with all predictors in the model, splitting the data and fitting separate models for each source habitat allowed the models to converge. However, AICc comparisons (with models fit using ML) indicated the random intercept model was preferred to the random slope and intercept model for the urban habitat colonies (ΔAICc > 5, with the random intercept model having the lower AICc value; Burnham, Anderson, & Huyvaert, 2010). Further, the random intercept model and the random slope and intercept model were not meaningfully different (ΔAICc < 2) for the rural habitat colonies, in which case we preferred the simpler random intercept model.

We used the Kenward–Roger *F*‐statistic approximation to assess the statistical significance of individual predictors in the model. To interpret the results of our statistical analyses, a significant effect of source habitat indicates evolutionary change in metabolic rate between urban and rural ants; a significant effect of test temperature indicates acute phenotypic plasticity in metabolic rate; and a significant interaction between source habitat and test temperature indicates evolved acute plasticity.

After fitting this model, we performed post hoc analyses with the *emmeans* function and library in R (Lenth, 2019) to explore the interaction between source habitat and test temperature. Specifically, for each source habitat we examined whether there was a difference in metabolic rate across the two test temperatures. Similarly, for each test temperature, we examined whether there was a difference in metabolic rate between the urban and rural source habitats.

We performed a series of complementary post hoc analyses to better understand the responses of colony‐level metabolic rate responses to temperature. First, we asked whether urban versus rural source habitats exhibited relatively greater variation in the slope or elevation of colony metabolic rate responses to temperature. Specifically, for each source habitat, we computed the coefficient of variation for colony metabolic rate at each of the two test temperatures and colony metabolic rate plasticity across the two test temperatures. Further, because mean thermal performance can trade off with plasticity in thermal performance in ectothermic species (Stillman, 2003), we also explored the relationship between mean metabolic rate and metabolic rate plasticity for urban and rural source habitats. We used a linear model with metabolic rate plasticity as the response and mean metabolic rate as the predictor. We performed separate models for urban and rural source habitats owing to issues with data separation between the two source habitats that precluded fitting a single model with an interaction between source habitat and mean metabolic rate. Although metabolic rate plasticity was uncorrelated with colony mass, mean metabolic rate was strongly correlated with colony mass (see Section 3). Therefore, in our analyses of the potential mean‐plasticity trade‐off for metabolic rate, we used the residuals from a linear model of metabolic rate as a function of colony mass to account for among‐colony variation in mass and its effects on mean metabolic rate. The computation of residuals was conducted separately for each source habitat.

#### 2.3.2 Running speed experiment

We fit a linear mixed effects model, again using the *lmer* function from the {lmerTest} library in R, to examine the effects of source habitat (urban or rural), test temperature (22°C, 32°C, or 42°C), distance interval (20, 40, 60, 80 or 100 mm), and their two‐ and three‐way interactions on individual running speed. To allow nonmonotonic relationships between running speed and test temperature or distance interval, we treated these predictors as factors. Because the data were modeled at the level of the individual rather than level of the colony as with metabolic rate, we updated the random effects structure from that used in our metabolic rate model to account for repeated measures on an individual across multiple test temperatures. Specifically, we included individual nested within colony nested within site as a random effect in the running speed model. We present the results of this nested random intercept model, as the random slope and intercept model did not converge and AICc comparison of models that treated distance interval and temperature as continuous indicated that the random intercept model was preferred to the random slope and intercept model (ΔAICc > 5, with the random intercept model having the lower AICc value). We used the Kenward–Roger *F*‐statistic approximation to assess the statistical significance of individual predictors in the model. The general interpretations of the significance of predictors is comparable to those for metabolic rate described above; for running speed, the additional predictor of distance interval can be interpreted as a measure of fatigue. Our analyses allowed us to assess whether ants became more fatigued over longer distances (slower running speed), and whether this relationship differed across test temperature, source habitat, or both.

To be able to explore and interpret running speed differences between the two source habitats at the same test temperatures as the metabolic rate experiment, we fit a thermal performance curve to the running speed data. This approach further allowed us to make inferences regarding the evolution of thermal performance curve shape in response to urban heat islands (McLean et al., 2005; Tüzün et al., 2017). As the estimation of thermal performance curves is often vastly improved with greater numbers of test temperatures (Angilletta, 2006), we included the lower and upper endpoints of coordinated movement, that is, colony mean CT_min_ and CT_max_ estimated from a previous laboratory common garden experiment (Diamond, Chick, Perez, Strickler, & Martin, 2018). From this experiment, we focused only the 25°C laboratory rearing treatment and those ants from urban and rural source habitats in Cleveland, Ohio, USA. The ant colonies assessed for CT_min_ and CT_max_ were from the same sites and populations as the running speed ant colonies (Table S1). There were a total of nine urban colonies and nine rural colonies (Table S1), each with 10 workers tested for CT_min_ and 10 workers tested for CT_max_. Ants were assigned a running speed of 0 mm/s for both CT_min_ and CT_max_.

We used nonlinear least squares to fit a curve to running speed responses across 5 temperatures including the three test temperatures and CT_min_ and CT_max_ endpoints of running speed performance. We fit separate thermal performance curves for urban versus rural source habitat ants. To avoid pseudoreplication, we computed colony mean running speed, pooled across the five distance intervals, and used these values to fit the thermal performance curves. To model running speed thermal performance curves, we used a standard insect thermal performance curve equation (Shi, Ge, Sun, & Chen, 2011) and estimated the parameter values with the *nls* function from the {nlme} library in R (Pinheiro, Bates, DebRoy, & Sarkar, 2019). We estimated separate curves for urban and rural ants. For each of these curves, we estimated 95% CIs using MCMC methods with the *predictNLS* function from the {propagate} library in R (Spiess, 2018).

Additionally, we used template mode of variation analysis to decompose variation in thermal performance curve shape between the urban and rural source habitats into shifts in the mean, height, and width of the curve (Izem & Kingsolver, 2005). These analyses quantify the percent of total variation in curve shape due to horizontal shifts (hotter‐colder), vertical shifts (faster‐slower), and shifts in width (specialist–generalist trade‐offs) plus an error term (unexplained variation). We performed two template mode of variation analyses. One analysis used the running speed data for the three test temperatures, and a second analysis incorporated the zero values for running speed at the location of the CT_min_ and CT_max_. All template mode of variation analyses were performed in MATLAB (R2019b) using the code of Izem and Kingsolver (2005).

## 3 RESULTS

### 3.1 Metabolic rate experiment

**FIGURE 2:**
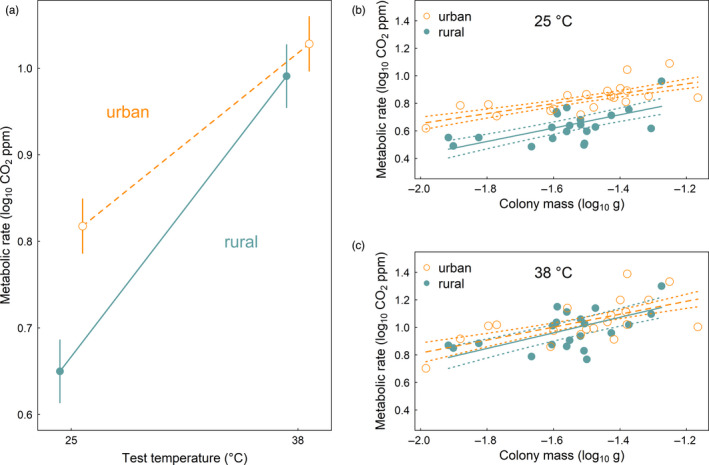
Evolutionary divergence of acorn ant metabolic rate across urban and rural source habitats. (a) Whole‐colony metabolic rate (log_10_ CO_2_ ppm) assessed at the laboratory rearing temperature, 25°C, and after a 2‐hr exposure to 38°C. Points represent estimated mean metabolic rate (±1 *SE*) of urban and rural colonies at the two test temperatures from a linear mixed effects model that accounts for the effects of colony mass and autocorrelation among colonies and sites. Metabolic rate is also presented as a function of colony mass (log_10_ g) for urban and rural colonies at the (b) 25°C test temperature and (c) 38°C test temperature. Predicted values (±1 *SE*) are shown in each panel

At 25°C, we found evidence of evolutionary divergence in whole‐colony metabolic rate between urban and rural source habitats. However, the pattern was opposite of our prediction: urban colonies exhibited higher metabolic rates compared with rural colonies (Figure 2a; Tables 1 and 2). Although there was a very weak trend for a similar pattern of divergence at 38°C, metabolic rates were statistically indistinguishable between urban and rural source habitats under these conditions (Figure 2a; Tables 1 and 2). Owing to the significant increase in metabolic rate of the urban colonies at 25°C and the nonsignificant divergence between urban and rural source habitats at 38°C, the acute plastic response to temperature was significantly different between source habitats. Specifically, the urban colonies exhibited diminished acute plasticity in metabolic rate compared with the rural colonies. Expressed in terms of the exponential temperature dependence coefficient, the urban colonies (*Q*_10_ = 1.47 ± 0.22) were 26% less sensitive to temperature than the rural colonies (*Q*_10_ = 1.85 ± 0.33).

**TABLE 1:** Model estimates, standard errors, test statistics, degrees of freedom, and *p*‐values (using the Kenward–Roger approximation) for the effects of source habitat, test temperature, and their interaction on metabolic rate. The rural habitat is the baseline level for the source habitat term. Colony mass is included as a covariate

Term	Estimate	*SE*	*F*	*ndf*	*ddf*	*p*
Log_10_ colony mass	0.457	0.0782	32.3	1	37.0	**<.0001**
Source habitat	0.168	0.0476	5.01	1	3.10	.108
Test temperature	0.341	0.0199	376	1	39.0	**<.0001**
Source habitat × test temperature	−0.130	0.0285	21.0	1	39.0	**<.0001**

Note

Significant *p‐*values are indicated in bold.

**TABLE 2:** Post hoc analyses of the interaction between source habitat and test temperature for metabolic rate

Contrast	Estimate	*SE*	*t*	*p*
*Source habitat differences given test temperature*				
Rural, 25°C ‐ urban, 25°C	−0.168	0.0475	−3.49	**.0279**
Rural, 38°C ‐ urban, 38°C	−0.0374	0.0475	−0.778	.483
*Test temperature differences given source habitat*				
Rural, 25°C ‐ rural, 38°C	−0.341	0.0199	−17.2	**<.0001**
Urban, 25°C ‐ urban, 38°C	−0.211	0.0204	−10.3	**<.0001**

Note

Significant *p‐*values are indicated in bold.

Colony mass had predictable effects on metabolic rate, such that metabolic rate increased with increasing colony mass (Figure 2b,c; Table 1). However, the relationship between colony mass and metabolic rate did not differ among source habitats or test temperatures (prior model constructs indicated nonsignificant interactions of colony mass with test temperature and with source habitat; for simplicity, we omitted interactions with colony mass and other predictors in our final model). Indeed, outside of our models of metabolic rate, we found no difference in colony mass between urban and rural source habitats (simple linear regression of log_10_ colony mass as a function of source habitat: *F*_1,39_ = 0.667, *p* = .419 for the effect of source habitat).

**FIGURE 3:**
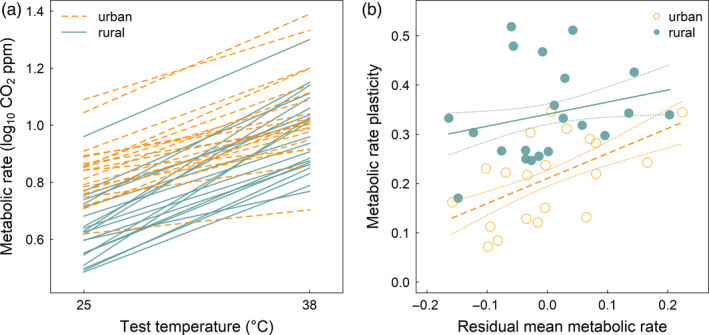
Colony‐level metabolic rate responses. (a) Per‐colony metabolic rate at 25°C and after a 2‐hr exposure to 38°C. Each line represents the colony reaction norm, that is, the plastic response of a given colony across the two test temperatures. (b) Relationship between metabolic rate plasticity (slope of the relationship between metabolic rate and test temperature for each colony) and mean metabolic rate among the two test temperatures. Due to effects of colony mass on mean metabolic rate (but not metabolic rate plasticity), mean metabolic rate is expressed as the residuals of the relationship between metabolic rate and colony mass. The simple linear regressions (predicted values ±1 *SE*) of metabolic rate plasticity as a function of residual mean metabolic rate are shown for the urban and rural source habitats

The individual colony reaction norms (plastic response across the two test temperatures) all trended positive, though there was considerable variation in the magnitude of the plastic response among colonies (Figure 3a). Further, the magnitude of variation in the plastic response was modestly higher in the urban colonies compared with the rural colonies: The coefficient of variation (CV) of the urban source habitat plasticity was 40.9, whereas the CV of the rural source habitat plasticity was 28.0. Importantly, the plastic response to temperature was not correlated with colony mass for either source habitat (*r*_urban_ = 0.280, *p* = .232; *r*_rural_ = 0.0819, *p* = .724). However, we did detect a significant positive correlation between colony mass and metabolic rate for each source habitat at each test temperature (*r*_urban,25_ = 0.690, *p* = .000762; *r*_rural,25_ = 0.579, *p* = .00595; *r*_urban,38_ = 0.625, *p* = .00324; *r*_rural,38_ = 0.546, *p* = .0105). Therefore, the interpretation of CV for metabolic rate of each source habitat at each test temperature includes a signal of colony mass. Because colony mass did not differ among urban and rural source habitats and colonies from both source habitats had similar relationships between metabolic rate and colony mass, our interpretation of relative differences in CV between urban and rural source habitats should be robust to colony mass effects. Here we found that the CV for metabolic rate at 25°C and at 38°C was similar for urban and rural source habitats (CV_urban,25_ = 13.0; CV_rural,25_ = 18.3; CV_urban,38_ = 15.1; CV_rural,38_ = 14.1). Thus, while we found greater variation in the plastic response of urban colonies, we found no difference between urban and rural source habitats in colony metabolic rate within each test temperature.

Finally, the relationship between mean colony metabolic rate and acute plasticity was different between ants from urban versus rural source habitats (Figure 3b). While mean colony metabolic rate was not a significant predictor of acute plasticity for ants from the rural source habitat (*F*_1,19_ = 1.16, *p* = .294, β = 0.245, SE = 0.227), there was a significant positive association between these traits for ants from the urban source habitat (*F*_1,18_ = 8.14, *p* = .0106, *β* = 0.507, *SE* = 0.178).

### 3.2 Running speed experiment

**FIGURE 4:**
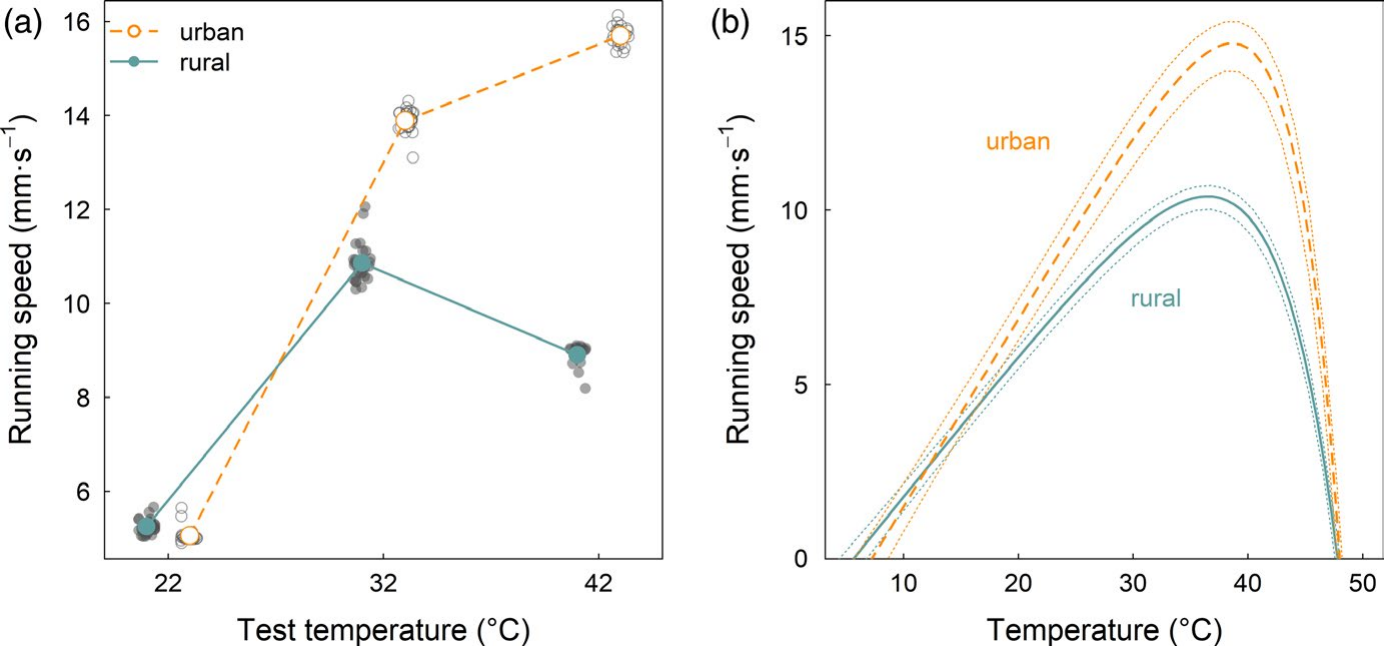
Evolutionary divergence of acorn ant running speed across urban and rural source habitats. (a) Estimated mean running speed of urban and rural source habitat ants at the three test temperatures (22°C, 32°C, and 42°C) from a linear mixed effects model that accounts for the effects of distance interval and autocorrelation among individuals, colonies, and sites. Means are offset slightly from their test temperatures to avoid overlap of points. Standard errors were sufficiently small to be obscured by the points representing the estimated means. In lieu of displaying standard errors, we provided the mean running speed for each colony, pooling across the level of individual and distance interval (smaller points, beneath the treatment × source habitat means). Colony means were jittered across the temperature axis to enhance their visibility. (b) Estimated thermal performance curves and 95% confidence intervals are shown for each source habitat

For the urban source habitat, increasing test temperature contributed to faster running speed (Figure 4a; Table 3). However, for the rural source habitat, ants ran fastest at 32°C compared with the cooler (22°C) and warmer (42°C) test temperatures. Within each test temperature, urban ants ran faster than rural ants at the two highest temperatures, 32°C and 42°C, whereas rural ants ran faster than urban ants at the lowest test temperature, 22°C (Table 4). Finally, we did not detect an effect of the distance ants ran on running speed, including no evidence of an interactive effect with source habitat or test temperature (Table 3).

**TABLE 3:** Test statistics and *p*‐values (using the Kenward–Roger approximation) for the effects of source habitat, test temperature, distance interval, and their interaction on running speed

Term	*F*	*ndf*	*ddf*	*p*
Distance interval	0.152	4	7,390	.962
Source habitat	6.05	1	45.5	**.0178**
Test temperature	2,920	2	7,390	**<.0001**
Distance interval × Source habitat	0.651	4	7,390	.626
Distance interval × Test temperature	1.80	8	7,390	.0726
Source habitat × Test temperature	2,380	2	7,390	**<.0001**
Source habitat × Test temperature × Distance interval	1.75	8	7,390	.0811

Note

Significant *p‐*values are indicated in bold.

**TABLE 4:** Post hoc analyses of the interaction between source habitat and test temperature (treated as a factor) for running speed

Contrast	Estimate	*SE*	*z*	*p*
*Source habitat differences given test temperature*				
Rural, 22°C ‐ urban, 22°C	0.191	0.0512	3.73	**.0002**
Rural, 32°C ‐ urban, 32°C	−3.03	0.0512	−59.2	**<.0001**
rural, 42°C ‐ urban, 42°C	−6.79	0.0512	−133	**<.0001**
*Test temperature differences given source habitat*				
Rural, 22°C ‐ rural, 32°C	−5.61	0.0328	−171	**<.0001**
Rural, 22°C ‐ rural, 42°C	−3.66	0.0328	−112	**<.0001**
Rural, 32°C ‐ rural, 42°C	1.95	0.0328	59.6	**<.0001**
Urban, 22°C ‐ urban, 32°C	−8.83	0.0322	−275	**<.0001**
Urban, 22°C ‐ urban, 42°C	−10.6	0.0322	−331	**<.0001**
Urban, 32°C ‐ urban, 42°C	−1.81	0.0322	−56.2	**<.0001**

Note

Significant *p‐*values are indicated in bold.

The template mode of variation analysis revealed the strongest support for shifts in the mean and width of the thermal performance curve for running speed between urban and rural source habitats. Between 53% and 77% of the total variance was explained by these two modes combined (ratios of sum of squares, RSS, for the 3‐temperature thermal performance curve: RSS_mean_ = 0.308, RSS_width_ = 0.459, RSS_height_ < 0.0001, RSS_error_ = 0.233; 5‐temperature thermal performance curve including CT_min_ and CT_max_ values: RSS_mean_ = 0.252, RSS_width_ = 0.278, RSS_height_ = 0.00110, RSS_error_ = 0.469). Specifically, for the urban source habitat, the location of the thermal optimum increased and the range of thermal performance narrowed, resulting in increased thermal specialization. There was little support for a vertical shift in the thermal performance curves between source habitats. Rather, the trend for urban source habitat ants to exhibit higher running speed than the rural source habitat ants at the two warmest test temperatures appeared to be captured by the shift toward a narrower performance breadth from the template mode of variation analysis.

To quantify the magnitude and direction of shifts in the thermal performance curve attributes, we used nonlinear least squares to perform the curve‐fitting and extracted shifts in the location of the thermal optimum, the shifts in peak running speed, and the breadth of thermal performance (Figure 4b). Our model estimated the thermal optimum for the rural source habitat to be 36.4°C, whereas the thermal optimum for the urban source habitat was estimated to be 38.8°C. At each source habitat's respective thermal optimum, urban ants ran 4.4 mm/s faster than the rural ants (the running speed at the rural optimum was 10.4 mm/s). Despite the urban source habitat ants exhibiting a higher thermal optimum and higher performance at the thermal optimum, their thermal range (high − low temperature values where running speed is zero: 48.0 − 7.21 = 40.8°C) and thermal breadth (upper temperature at 80% maximum performance − lower temperature at 80% maximum performance: 43.9 − 29.4 = 14.5°C) were narrower compared with the thermal range and breadth of the rural source habitat ants (thermal range: 47.8 − 5.59 = 42.2°C; thermal breadth: 42.6 − 26.7 = 15.9°C). This result was driven by the urban source habitat ants exhibiting a relatively modest gain in their upper limit on running speed while at the same time losing a considerable amount of low temperature running speed performance compared with the rural source habitat ants.

The estimation of the thermal performance curve for running speed further allowed us to estimate source habitat differences in running speed at the two metabolic rate test temperatures (25°C and 38°C). Our model predicted that urban ants would run faster than rural ants at each of the two metabolic rate test temperatures: 9.52 versus 7.67 mm/s at 25°C and 14.8 versus 10.3 mm/s at 38°C.

## 4 DISCUSSION

The expectation of countergradient variation in metabolic rate of ectotherms across temperature clines has been called “one of the most controversial hypotheses in physiological ecology” (White, Alton, & Frappell, 2012); however, the ability of organisms to metabolically compensate for variation in climate has never been more important given ongoing trends of recent climatic warming (Dillon et al., 2010; Moffett et al., 2018). Evidence of adaptive contemporary evolution of temperature‐sensitive traits is growing (De Meester et al., 2018), yet there are few empirical tests of compensatory evolution of metabolic rate in response to warming over contemporary timescales.

To both better understand the fundamental question of the effects of temperature on the evolution of metabolic rate as well as the more applied question of the potential for populations to mount compensatory evolutionary responses to human‐induced environmental warming, we performed an urban evolution common garden study. We used urban and rural populations of acorn ants to explore contemporary evolution of metabolic rate and locomotor performance in response to an urban heat island. Contrary to our expectation, we found evidence of the evolution of higher metabolic rate in urban ants compared with rural ants when tested at 25°C. While our study lends further support to the evidence for contemporary evolution of metabolic rate, the response of urban acorn ants is potentially maladaptive. We additionally found evidence of evolutionary divergence between urban and rural ants in locomotor performance such that urban ants exhibited greater running speed at warm test temperatures, but slower speed at a cool test temperature. This shift in locomotor performance at high temperatures could translate into improved resource acquisition in urban environments and might compensate for the increased metabolic demand of the urban ants. As the climate continues to warm through expansion of cities and their urban heat island effects as well as global climate change, our study suggests that understanding the potential for contemporary evolution in multiple thermal physiological traits, and whether those changes are adaptive or maladaptive, will be crucial for forecasting population persistence.

### 4.1 Metabolic rate

The evidence is relatively mixed for countergradient variation in metabolic rate across biogeographic clines in temperature (DeLong et al., 2018). Terrestrial systems appear to be more likely to show positive support for the pattern compared with aquatic systems (Schaefer & Walters, 2010; Sørensen, White, Duffy, & Chown, 2018). And indeed the pattern was weakly supported in a global synthesis of results from insect systems (Addo‐Bediako et al., 2002). However, there are many insect and other arthropod counterexamples as well (Lardies, Bacigalupe, & Bozinovic, 2004; May et al., 2018). In our study of acorn ants, rather than finding the expected pattern of countergradient variation, or even a lack of relationship between metabolic rate and temperature, we instead found a significant pattern of cogradient variation. Specifically, at 25°C, we found evidence of the evolution of higher metabolic rate in warmer‐climate urban acorn ant colonies compared with cooler‐climate rural colonies (Figure 2a). This result runs counter to biogeographic patterns among several ant species that show negative relationships between metabolic rate and habitat temperature across latitude and elevation (MacKay, 1982; Nielsen, Elmes, & Kipyatkov, 1999; Shik, Arnan, Oms, Cerdá, & Boulay, 2019).

Our results also run counter to the handful of contemporary evolution studies that have explored whole‐organism metabolic rate responses to climatic variation in temperature. Evolution of lower metabolic rate in response to warming has been shown for phytoplankton in an experimental evolution study (Padfield et al., 2016); for threespine stickleback in a comparative study of fish exposed to natural conditions versus artificially warmed conditions within the last half century (Pilakouta et al., 2020); and for mosquitofish introduced to springs of varying temperatures over the last century (Moffett et al., 2018). In *Daphnia*, the evolution of lower baseline activity levels of enzymes important for stress physiology and energy metabolism including antioxidant defense enzymes has been specifically found across urbanization clines (Brans, Stoks, et al., 2018). Although this study was not specifically focused on temperature, the urban heat island effect is often a major part of generalized urbanization impacts, particularly in this system (Brans, Engelen, Engelen, Souffreau, & De Meester, 2018), so it is plausible that the evolution of these enzymatic responses are at least in part due to urban shifts in temperature.

Why then should acorn ants evolve a higher metabolic rate in warmer urban environments? Although there has been much focus in the literature on the possibility of evolutionary rescue from the impacts of environmental change, including global climate change and urbanization (Carlson, Cunningham, & Westley, 2014), recent work has highlighted the importance of maladaptive responses (Brady et al., 2019; Diamond & Martin, 2020). Most benignly, maladaptive responses could refer to simply not being able to keep pace with the magnitude and rate of environmental change (Radchuk et al., 2019); however, maladaptive responses can also manifest as responses in the opposite direction to cope with environmental change (Dayananda & Webb, 2017; Hale, Morrongiello, & Swearer, 2016; Van Dyck, Bonte, Puls, Gotthard, & Maes, 2015). Assuming that the evolution of lower metabolic rate in warmer climates is adaptive to limit costs and potential damage under high temperature conditions (Moffett et al., 2018), the evolution of higher metabolic rate in urban acorn ants would appear to be maladaptive. There are a number of potential explanations for why the metabolic rate response might be maladaptive. As one of the more likely explanations, it is possible that metabolic rate is correlated with other traits, such as heat tolerance, that show adaptive evolution to cities in the acorn ant system (Diamond, Chick, Perez, Strickler, & Martin, 2018). Further, although we selected our study sites to minimize confounding variables with temperature effects, evolutionary responses to other stressors such as environmental pollutants which can exhibit cross‐tolerance or trade‐offs with metabolic rate and thermal tolerance might also play a role (Sinclair, Ferguson, Salehipour‐shirazi, & MacMillan, 2013). Alternatively, the evolution of higher metabolic rate could be adaptive in other ways, for example, by speeding development (Penick, Diamond, Sanders, & Dunn, 2017) and allowing colonies to produce alates (reproductive ants) earlier in the year, with improved access to food and nest resources (Chick, Strickler, Perez, Martin, & Diamond, 2019). Although our data suggest that the metabolic rate response is in the opposite (maladaptive) direction based on both biogeographic comparative work and contemporary evolution studies, future research aimed at collecting data on acorn ant colony fitness will be important for interpreting the longer‐term consequences of the evolution of higher metabolic rate in the urban environment.

In the meantime, we can at least speculate on how organisms with elevated metabolic rate might incur fitness costs. The rate of living hypothesis provides one possible answer. Under this hypothesis, the production of reactive oxygen species from metabolism is expected to lead to greater oxidative damage (Monaghan, Metcalfe, & Torres, 2009). In turn, several fitness components might be negatively affected, including survival via reduced life span, and mating success and fecundity via poor body condition (*e.g*., Boyce, Mouton, Lloyd, Wolf, & Martin, 2020). Further, physiological responses to cities might also be associated with trade‐offs among fitness components, such as in side‐blotched lizards that show enhanced fecundity but reduced survival in response to urbanization (Lucas & French, 2012). For the acorn ant system in particular, the negative fitness consequences will be most important for the queen as the sole resident reproductive member of the colony; however, since the workers indirectly determine the queen's condition through their ability to forage and tend the nest, reductions in, for example, worker longevity, could nonetheless contribute to reductions in fitness.

By contrast with the results at the 25°C test temperature, we found that urban and rural acorn ant colonies had statistically indistinguishable metabolic rates at the 38°C test temperature (Figure 2a). Owing to the strong population divergence at 25°C and lack of divergence at 38°C, urban ants appear to have less acute plasticity in metabolic rate than rural ants. This result contrasts with results from a global synthesis of metabolic rate of ectotherms across latitudinal clines in temperature, where greater plasticity in metabolic rate was observed in warmer, less thermally variable environments (Seebacher, White, & Franklin, 2015). As the authors note, this result was unexpected, and so the diminished plastic response of urban acorn ant metabolic rate is actually more in line with the expectation arising from reduced diurnal temperature variance in cities (Imhoff, Zhang, Wolfe, & Bounoua, 2010). Though, interestingly, the temperature profiles for the acorn ant microclimates do not show much evidence of reduced diurnal temperature variance (Figure 1). Further, while any changes in diurnal temperature variance would be largely experienced by acorn ants within the nest environments, foragers might experience different conditions. In urban acorn ants, foragers encounter greater spatial variation in temperature in cities as they move across thermally heterogeneous landscapes generated by reduced canopy cover and altered habitat structure (Diamond, Chick, Perez, Strickler, & Zhao, 2018). Thus, how spatio‐temporal thermal variability shapes metabolic rate plasticity of acorn ants is somewhat unclear at this stage.

On this theme, it is worth considering the basis for the differences in metabolic rate plasticity across the urban and rural acorn ants. It is possible the urban acorn ants are becoming canalized for metabolic rate. Certainly we found relatively high amounts of variation in metabolic rate plasticity among urban ants at the colony level (Figure 3a) suggesting at least the potential for variation on which selection might act. Though as discussed above, it is unclear whether metabolic rate is the target of selection or simply a correlated response via selection on other traits. In addition, it is important to consider what the 38°C test temperature means to urban versus rural acorn ants. Over the time interval between mid‐June and mid‐July for which we have available temperature data within ant nest microsites, there were only 2 days (out of 30) where temperatures exceeded 38°C in the rural habitat, whereas there were 20 days (again, out of 30) where temperatures exceeded 38°C in the urban habitat (Figure 1; Diamond, Chick, Perez, Strickler, & Martin, 2018). Thus while 38°C might be relatively common for the urban ants and is below the estimated thermal optimum for locomotor performance from this study, it is infrequent for the rural ants and exceeds the estimated thermal optimum for locomotor performance (Figure 4b), indicating a possible stress response in metabolic rate for rural ants.

Although we have thus far considered responses of metabolic rate at each temperature and the acute plastic response independently, for a number of ectothermic species, mean or basal thermal performance trades off with plasticity (Stillman, 2003). Specifically, organisms with higher mean thermal performance are expected to exhibit a diminished plastic response. This pattern is thought to arise from constraints on expanding the range of thermal performance at already high performance values, that is, that there will be less variation as the upper limit is approached (Somero, Lockwood, & Tomanek, 2017). We found no association between mean metabolic rate and metabolic rate plasticity for the rural ants which is not wholly unexpected, as there is a fair amount of variation in the magnitude of the trade‐off among different species within the same study system (Armstrong, Tanner, & Stillman, 2019) and broad compilations of data across many ectothermic species show little evidence of such a trade‐off (Gunderson & Stillman, 2015). Surprisingly, we found a significant positive association between these traits for the urban ants (Figure 3b). Certainly positive associations are known for some select taxa (Verberk, Calosi, Spicer, Kehl, & Bilton, 2018), but in the case of acorn ants, we have divergent responses within the same species across recently isolated populations. In novel environments such as cities, positive associations between traits that would otherwise exhibit trade‐offs could be expected if those organisms that are able to survive and reproduce in the novel environments exhibit jack‐of‐all trades phenotypes until selection has an opportunity to refine this variation (Service & Rose, 1985). However, given the ambiguity surrounding whether greater mean metabolic rate and greater plasticity in metabolic rate are beneficial for urban acorn ants, the exploration of such relationships awaits future studies linking trait values with other aspects of performance and fitness.

The one way in which acorn ants met our expectations for metabolic rate was through colony mass effects on metabolic rate. We found that colony mass had positive effects on metabolic rate (Figure 2b,c) as observed broadly across a diverse suite of taxa (White et al., 2019). Importantly, however, we found that the relationship between metabolic rate and colony mass was not significantly different among urban and rural source habitats.

### 4.2 Running speed

Much in the same way that we found unexpected evolutionary responses of metabolic rate in urban acorn ants, so too did urban ants yield surprising results for running speed, our measure of locomotor performance. Although urban ants ran slower than rural ants at the coolest test temperature of 22°C, they ran considerably faster than rural ants, on the order of several mm/s to more than double the rate of rural ant running speed, at the two highest test temperatures of 32°C and 42°C (Figure 4a). This pattern was also reflected in the shape of the fitted thermal performance curve and the template mode of variation analysis that allowed us to decompose variation in curve shape between urban versus rural source habitats. We found evidence of an increased thermal optimum of the urban ants and increased thermal specialization such that urban ants exhibited a narrower breadth of performance but comparatively high performance at high temperatures (Figure 4b). Both of these shifts are supported by theory (Angilletta, 2009; Goulet, Thompson, Michelangeli, Wong, & Chapple, 2017).

Interestingly, for studies that were able to measure multiple traits or multiple species for the evolution of thermal performance curves in response to urban heat islands, a range of different shifts in thermal performance curve shape were detected. For example, two species of chitinolytic fungi showed evidence of shifts in the thermal performance curve breadth (specialist–generalist trade‐off), whereas another two species showed evidence of vertical shifts wherein urban populations grew faster across all temperatures (McLean et al., 2005). In addition, a damselfly species showed evidence of vertical shifts for both survival and development time, though the shift was in the cogradient direction for survival, but countergradient direction for development time (Tüzün et al., 2017). This variation in the type of contemporary evolution of performance in response to urbanization is also reflected in other studies, but which do not necessarily estimate curve shape. For example, water fleas (Brans et al., 2017; Brans, Stoks, et al., 2018) and the acorn ant system that is the focus of the work presented here (Chick et al., 2019; Diamond, Chick, Perez, Strickler, & Martin, 2018) exhibit a suite of co‐ and countergradient responses across physiological and life history traits in response to urbanization. With increased numbers of developmental and test temperatures, the evolution of curve shape for a number of traits could also be explored in these and other systems. In general, cities could be good candidates for exploring the evolution of thermal performance curves (Tüzün & Stoks, 2018), particularly given the widespread disconnect between theoretical expectations of the evolution of thermal performance curves and results from empirical studies (Kingsolver, 2009) and the potential for high levels of replication within and among cities.

With respect to our specific study system, because foraging ants are the only mechanism of resource intake for acorn ant colonies, the success or failure of the colony—specifically, the survival of the queen and her ability to acquire sufficient resources for the production of new cohorts of sterile nest workers, foragers and reproductive (nonsterile) ants (Ingram, Pilko, Heer, & Gordon, 2013)—is contingent upon worker foraging ability (Traniello, 1989). Enhanced locomotor function such as via increased running speed can be important for tasks such as nest site discovery, but it can also allow scouts to find resources faster and allow ants to return to the nest faster with the acquired resource (Heinrich, 2013; Lach et al., 2010). Owing to the small body size of acorn ants and the low number of workers per colony which serve to limit predation and competition, running speed appears to be most important for scouting new nest site locations and acquiring food resources (Fellers, 1987; Pratt, 2008). Although the remarkably consistent plastic effects of temperature on running speed in ants have been known from the 1920s (Shapley, 1920), the evolution of running speed in response to temperature has received less attention. Granted, a number of studies have demonstrated interspecific variation in running speed; however, a considerable portion of this variation is attributable to species differences in body size (Hurlbert et al., 2008; Yanoviak, Silveri, Stark, Stan, & Levia, 2017). Acorn ants show no evolved divergence (or even thermal plasticity) in body size across urban and rural populations from multiple cities (Yilmaz et al., 2019); thus, the population divergence in running speed of this species is independent of body size.

Beyond providing another data point for studies showing evidence of contemporary evolution of thermal performance traits in cities, perhaps the most important consequence of the faster running speed of urban ants is its potential feedback on metabolic rate. While urban acorn ants evolved a higher metabolic rate (Figure 2), they also exhibited an evolved increase in the locomotor performance trait of running speed (Figure 4), which could indicate greater potential for resource acquisition (Leonard & Herbers, 1986). In effect, the faster running speed of urban acorn ants could facilitate a greater influx of resources and possibly compensate for increased metabolic demand. Climatic warming at the global scale is expected to lead to metabolic meltdown, wherein metabolic rate exceeds resource acquisition and allocation (Huey & Kingsolver, 2019). However, such predictions are based on the lack of compensatory responses. In the case of the acorn ants, while the evolution of higher metabolic rate in the city appears to be maladaptive, the evolution of faster resource acquisition could provide an indirect route of compensation. Of course there are other ways to alter metabolic rate beyond the rate of resource acquisition. For example, nutrient excretion rates and nutrient recycling can alter metabolic rate (Moffett et al., 2018). And different available resource pools; a preference for consuming different types of food; or the perception of low‐food conditions can also alter metabolic rate (Norin & Metcalfe, 2019).

Although we provided acorn ants with the same food resources—sugar water, a carbohydrate source, and mealworms, a lipid and protein source—when reared in the laboratory, it is possible acorn ants selected different food items, or different ratios of food items that altered their metabolic rate in our experiment. However, because we reared ants under a common garden design until a new, laboratory‐born cohort of workers was generated, any changes in behavioral preferences for different food types would be evidence of an evolved response. Therefore, different nutrient ratios would provide a mechanism for divergence in metabolic rate rather than an alternative explanation of our results in the strict sense. In the field, it is possible that there are ecological effects of resource quality and availability on metabolic rate; however, because we selected urban sites that were forest patches embedded within an impervious surface matrix, habitats are quite similar across urban and rural sites, save the elevated temperature in urban sites. Further, these habitats have similar abundance, if not diversity, of detritus resources that are the primary food source of acorn ant colonies (Perez & Diamond, 2019).

Finally, it is important to consider why acorn ants do not show population differences in activity within the metabolic rate experiment, but do so in the running speed experiment across the same temperature range. Here, context is key. Acorn ants exhibit relatively little activity within the nest environment (Herbers & Cunningham, 1983; Herbers & Johnson, 2007), and the respirometry chamber with the whole‐colony intact replicates the nest environment. By contrast, when ants were tested for running speed, foragers were isolated from the colony, and prompted to run down the testing track. Because ants behave differently in different contexts, we only observed evolutionary divergence in foraging‐related activity, not nest activity. If we were able to test metabolic rate of individual ants in a foraging context, it is plausible that any observed divergence in metabolic rate would be driven in some part by population divergence in activity.

### 4.3 Limitations and future directions

Our study provides evidence of the evolution of metabolic rate and resource acquisition in urban acorn ants. Although this research helps to build a foundation for the evolution of whole‐organism metabolic traits in response to warming over contemporary timescales, there are many areas for improvement and further exploration. Ideally, we would be able to test laboratory‐born workers and queens, not just laboratory‐born workers and field‐caught queens, for the assessment of whole‐colony metabolic rate to better understand the potential role of transgenerational plasticity. Previously, we have shown that the evolution of thermal tolerance traits of acorn ants in response to urbanization is not driven by maternal effects of queens (Martin et al., 2019); however, it is possible maternal effects could have a detectable influence on other traits such as metabolic rate. Though it is still unclear how large this effect might be. Secondly, the genetic architecture of metabolic rate and locomotor performance is not known from our current experimental design, as the same colonies were not tested for both metabolic and locomotor traits. As both traits are nondestructive, the genetic correlations among these traits could be assessed. Thirdly, the nature of the plastic response of metabolic rate to temperature in urban and rural source habitats could be characterized more completely. In particular, testing metabolic rate plasticity under a broader range of developmental and test temperatures (e.g., Maclean et al., 2017) is necessary to quantify the shape of the thermal performance curve for this trait and understand how urban and rural ants have diverged. Finally, although our study demonstrates that rapid evolution of metabolic traits is possible in response to urban heat islands, an important next step is to explore this question across multiple cities and multiple taxa. Because cities represent a globally replicated warming experiment, they can be used to test the potential for parallel evolution of metabolic traits under climatic warming (e.g., Campbell‐Staton et al., 2020).

## AUTHOR CONTRIBUTIONS

JSW and LDC performed metabolic rate measurements and analyses. LDC performed running speed measurements. SED performed statistical models. SED and LDC wrote the first draft. All authors contributed to revising the manuscript.

## Supporting information

Supplementary Material

## Data Availability

Data for this study are available at the Dryad Digital Repository: https://doi.org/10.5061/dryad.5mkkwh73v.
