# Supplementary material for: Pedal to the metal: Cities power evolutionary divergence by accelerating metabolic rate and locomotor performance
Source: Evol Appl. 2020 Sep 25;14(1):36–52. doi: 10.1111/eva.13083 (PMC7819567; doi:10.1111/eva.13083)
Supplement: Supplementary file 1 — Supplementary Material [file EVA-14-36-s001.pdf]

## Supplementary Information

### Methods and results for activity within respirometry chambers

We aimed to test: (1) whether ants from the two source habitats (urban or rural) exhibited different activity responses when placed into respirometry chambers within growth chambers held at 25 and 38 °C, and (2) whether ants had generally high or low activity levels within respirometry chambers. Note that the ants from this experiment were placed within respirometry chambers, but were unable to be tested for metabolic rate.

We first tested whether the colonies assessed for activity were of comparable size to those were able to test for metabolic rate. A simple linear model of total colony size (the number of workers, brood and queens) as a function of whether the colonies were used in the activity experiment or the metabolic rate experiment indicated no difference in colony size between the experiments (Fig. S1; $F_{1,47} = 0.00810$, $P = 0.929$).

For the assessment of activity, we quantified the number of workers per colony moving around the respirometry chamber. We did this over a span of 20 minutes with 11 observation time points of 10 seconds each occurring every 2 minutes. We had a total of 176 observations across 8 colonies (Table S1). We constructed a linear mixed effects model with the number of active workers per colony as the response variable, and source habitat, test temperature and observation time point as predictor variables. As the response variable was not normally distributed and contained zeroes, we natural log (+1) transformed this variable prior to fitting the model. For the predictor variables, we fit up to the three-way interaction term, including all two-way interactions and main effects. We included the total number of workers per colony as a covariate to account for potential behavioral differences or sampling effects of larger colonies. We also included colony identity as a random intercept, as colonies were measured repeatedly across the two test temperatures and observation time points. For assessing statistical significance of the predictors, we used the Kenward-Roger *F*-statistic approximation.

Our model revealed no significant effects of any of the predictor variables on acorn ant activity within the respirometry chamber. Most importantly, urban and rural source habitat colonies had comparable levels of activity when housed within the respirometry chambers, suggesting that population differences in activity are unlikely to provide a basis for divergence in metabolic rate (Table S3).

Further, we found activity levels of acorn ants to be overall quite low. For example, the mean proportion of workers active at any given observation time point (and from any source population or test temperature) was 0.12 (Fig. S2). We did observe a marginal trend towards increased activity at the warmer test temperature as might be expected, however, this result was not statistically significant (Table S3).

**Table S1.** Colony collection sites for acorn ants. The percent developed impervious surface area (ISA) for the 120 m buffer around each site and the numbers of colonies tested per each site are provided.

| Experiment | Habitat type | Site | Longitude | Latitude | ISA | Number of colonies |
|---|---|---|---|---|---|---|
| Metabolic rate (metabolic rate data from the test temperatures 25 and 38 °C) | Rural | CWRU Farm | -81.423 | 41.499 | 0 | 12 |
| | | Holden Arboretum | -81.313 | 41.609 | 0 | 9 |
| | Urban | CWRU Campus | -81.609 | 41.503 | 61 | 7 |
| | | Doan Brook | -81.614 | 41.509 | 42 | 7 |
| | | Ambler Park | -81.606 | 41.498 | 40 | 3 |
| | | Forest Hill | -81.571 | 41.521 | 46 | 3 |
| Metabolic rate (activity data from the test temperatures 25 and 38 °C) | Rural | CWRU Farm | -81.423 | 41.499 | 0 | 2 |
| | | Holden Arboretum | -81.313 | 41.609 | 0 | 2 |
| | Urban | CWRU Campus | -81.609 | 41.503 | 61 | 2 |
| | | Ambler Park | -81.606 | 41.498 | 40 | 2 |
| Running speed (running speed data from the test temperatures 22, 32 and 42 °C) | Rural | CWRU Farm | -81.423 | 41.499 | 0 | 16 |
| | | Holden Arboretum | -81.313 | 41.609 | 0 | 10 |
| | Urban | Acacia Reservation | -81.490 | 41.502 | 51 | 5 |
| | | Shaker Lake | -81.574 | 41.485 | 40 | 6 |
| | | Doan Brook | -81.614 | 41.509 | 42 | 6 |
| | | Ambler Park | -81.606 | 41.498 | 40 | 5 |
| | | Forest Hill | -81.571 | 41.521 | 46 | 5 |
| Running speed ($CT_{min}$ and $CT_{max}$ data from Diamond et al., 2018* to approximate the temperatures when running speeds are 0) | Rural | CWRU Farm | -81.423 | 41.499 | 0 | 4 |
| | | Holden Arboretum | -81.313 | 41.609 | 0 | 5 |
| | Urban | Doan Brook | -81.614 | 41.509 | 42 | 7 |
| | | Ambler Park | -81.606 | 41.498 | 40 | 2 |

* Diamond, S. E., Chick, L. D., Perez, A., Strickler, S. A., & Martin, R. A. (2018). Evolution of thermal tolerance and its fitness consequences: Parallel and non-parallel responses to urban heat islands across three cities. *Proceedings of the Royal Society B*, *285*, 20180036.

**Table S2.** Estimates (with standard errors), *F*-statistics, and *P*-values for comparisons of colony demographics (brood, workers, queens) between urban and rural acorn ant colonies censused at the time metabolic rate and running speed assays were performed. Estimates are expressed as the difference between rural (baseline) and urban source habitats.

| **Experiment** | **Response** | **Estimate** | **SE** | ***F*** | ***ndf*** | ***ddf*** | ***P*** |
|---|---|---|---|---|---|---|---|
| Metabolic rate | Brood | -0.283 | 5.92 | 0.00230 | 1 | 39 | 0.962 |
| | Workers | 0.588 | 5.02 | 0.0138 | 1 | 39 | 0.907 |
| | Queens | -0.139 | 0.138 | 1.00 | 1 | 39 | 0.323 |
| Running speed | Brood | 7.69 | 4.51 | 2.90 | 1 | 51 | 0.0945 |
| | Workers | 7.78 | 7.41 | 1.10 | 1 | 51 | 0.299 |
| | Queens | -0.155 | 0.0861 | 3.26 | 1 | 51 | 0.0771 |

**Table S3.** Results of the linear mixed effects model examining the effects of source habitat, test temperature and observation time point on the number of active workers per colony in the respirometry chambers.

| **Term** | ***F*** | ***ndf*** | ***ddf*** | ***P*** |
|---|---|---|---|---|
| Total number of workers in the colony | 0.0408 | 1 | 5 | 0.848 |
| Source habitat | 0.0944 | 1 | 84.2 | 0.759 |
| Test temperature | 3.06 | 1 | 126 | 0.0826 |
| Observation time point | 0.946 | 10 | 126 | 0.494 |
| Source habitat × Test temperature | 1.33 | 1 | 126 | 0.251 |
| Source habitat × Observation time point | 0.274 | 10 | 126 | 0.986 |
| Test temperature × Observation time point | 1.12 | 10 | 126 | 0.351 |
| Source habitat × Test temperature × Observation time point | 0.360 | 10 | 126 | 0.961 |

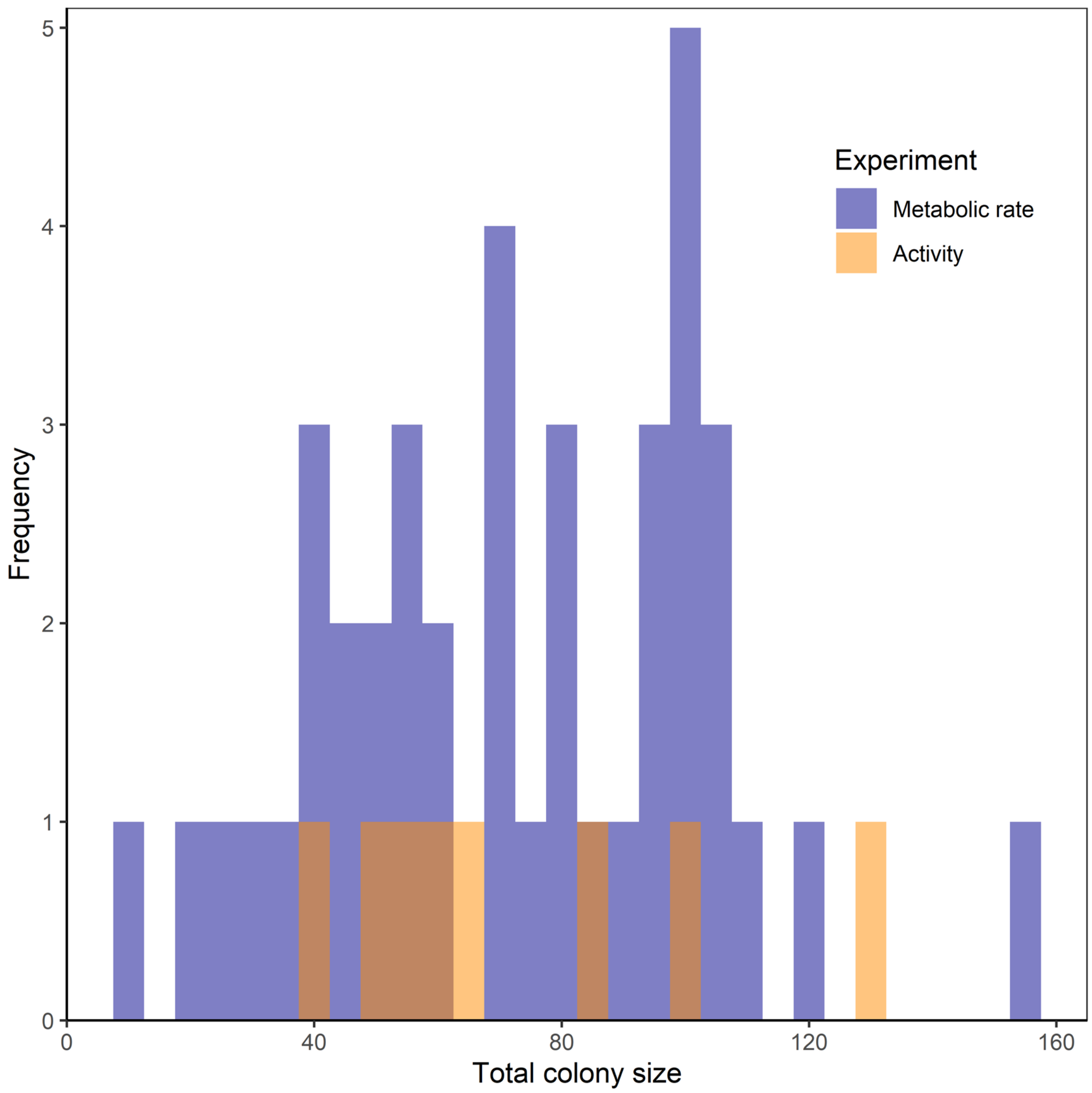


**Figure S1**. Histogram of the total colony size (number of workers, brood, and queens) that were part of the metabolic rate experiment (blue bars) or the activity experiment (orange bars).

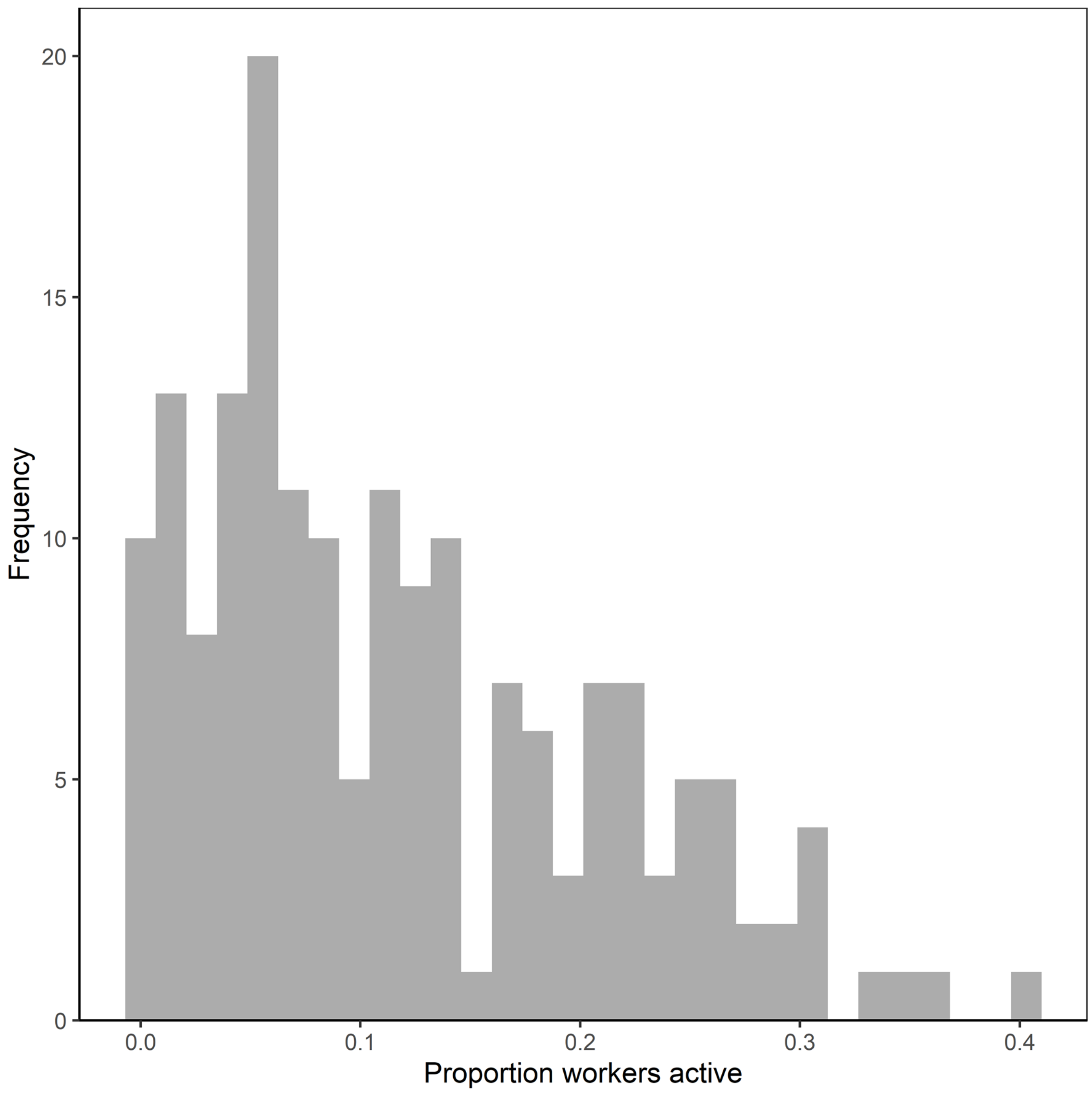


**Figure S2.** Histogram of the proportion of workers active within a colony across all source populations, test temperatures and observation time points.

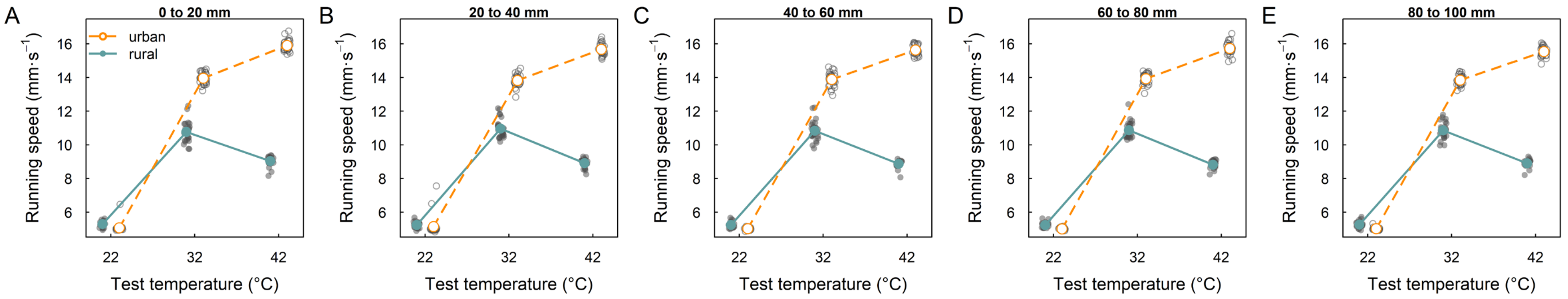


**Figure S3**. Estimated mean running speed of urban and rural source habitat ants at the three test temperatures (22, 32 and 42 °C) from linear mixed effects models that account for autocorrelation among individuals, colonies, and sites. Separate models were performed for each distance interval. Means are offset slightly from their test temperatures to avoid overlap of points. Standard errors were sufficiently small to be obscured by the points representing the estimated means. In lieu of displaying standard errors, we provided the mean running speed for each colony. Colony means were jittered across the temperature axis to enhance their visibility. Results are presented separately for each of the five distance intervals, including: (A) 0 to 20 mm; (B) 20 to 40 mm; (C) 40 to 60 mm; (D) 60 to 80 mm; and (E) 80 to 100 mm.
